# Predictors of In-Hospital Mortality in Surgical Wards: A Multivariable Retrospective Cohort Analysis of 2,800,069 Hospitalizations

**DOI:** 10.1007/s00268-020-05841-3

**Published:** 2020-10-26

**Authors:** Magdalena Walicka, Agnieszka Tuszyńska, Marcin Chlebus, Yaroslav Sanchak, Andrzej Śliwczyński, Melania Brzozowska, Daniel Rutkowski, Monika Puzianowska-Kuźnicka, Edward Franek

**Affiliations:** 1 Department of Internal Diseases, Endocrinology and Diabetology, Central Clinical Hospital MSWiA, ul. Woloska 137, 02-507 Warsaw, Poland; 2 Department of Quantitative Finance, Faculty of Economic Sciences, University of Warsaw, ul. Dluga 44/50, 00-241 Warsaw, Poland; 3 Warsaw Medical University, ul. Zwirki i Wigury 61, 02-091 Warsaw, Poland; 4 National Health Fund, ul. Grojecka 186, 02-390 Warsaw, Poland; 5 Department of Human Epigenetics, Mossakowski Medical Research Centre, Polish Academy of Sciences, ul. Pawinskiego 5, 02-106 Warsaw, Poland; 6 Department of Geriatrics and Gerontology, Medical Centre of Postgraduate Education, Ceglowska 80, 01-809 Warsaw, Poland

## Abstract

**Background:**

Identifying prognostic factors that are predictive of in-hospital mortality for patients in surgical units may help in identifying high-risk patients and developing an approach to reduce mortality. This study analyzed mortality predictors based on outcomes obtained from a national database of adult patients.

**Materials and methods:**

This retrospective study design collected data obtained from the National Health Fund in Poland comprised of 2,800,069 hospitalizations of adult patients in surgical wards during one calendar year. Predictors of mortality which were analyzed included: the patient’s gender and age, diagnosis-related group category assigned to the hospitalization, length of the hospitalization, hospital type, admission type, and day of admission.

**Results:**

The overall mortality rate was 0.8%, and the highest rate was seen in trauma admissions (24.5%). There was an exponential growth in mortality with respect to the patient’s age, and male gender was associated with a higher risk of death. Compared to elective admissions, the mortality was 6.9-fold and 15.69-fold greater for urgent and emergency admissions (*p* < 0.0001), respectively. Weekend or bank holiday admissions were associated with a higher risk of death than working day admissions. The “weekend” effect appears to begin on Friday. The highest mortality was observed in less than 1 day emergency cases and with a hospital stay longer than 61 days in any type of admission.

**Conclusion:**

Age, male gender, emergency admission, and admission on the weekend or a bank holiday are factors associated with greater mortality in surgical units.

## Introduction

The number of surgical interventions is increasing. Weisser et al. approximated that around 312.9 million surgical procedures occurred in 2012, equating to an increase of about one third over an 8 year period [1]. Although surgical care prevents many deaths, complications and mortality are unfortunately inherent to such care.

In-hospital mortality rate is one of the indicators of the quality of health care. Numerous papers regarding in-hospital mortality have been published, however, they usually focus on deaths related to specific diseases. For example, Mazzeffi et al. analyzed all cardiac surgeries over an 8 year period in a single center and reported 3.4% in-hospital mortality rate [2]. In another study, Pucciarelli et al. assessed in-hospital mortality in patients who underwent surgical procedures for colorectal cancer, based on data derived from the National Italian Hospital Discharge Dataset [3]. Studies evaluating general in-hospital deaths are limited to small populations in single hospitals [4–6] or to specific age groups [7]. Other studies, while analyzing large or very large populations, do not distinguish surgical and non-surgical deaths [8–14].

Surgeons assessing the perioperative risk need to be aware that the risk of surgery-related complications and mortality depend on factors including those associated with the type of procedure [15–17]. The potential risk factors that should be considered are: quality of health care (the type of health care system organization and the mode of financing [18]), hospital type and volume [19–21], adherence to use of safety protocols [22], number of patients admitted to the intensive care unit as well as patient-related factors such as socioeconomic status [23], co-morbidity, frailty and last, but not least, patients’ age [24–26]. Important risk factors of in-hospital mortality are also emergencies and the duration of surgery [27]. However, there are no studies assessing predictors of in-hospital mortality after all types of surgeries in a very large population. Therefore, the aim of this investigation was to discern the prognostic factors predictive of in-hospital mortality in a nationwide sample of patients hospitalized in surgical wards.

## Material and methods

Statistics that were collected for the year 2014 in the database of the National Health Fund (NHF) were gathered for analysis. The NHF is a public organization which finances medical procedures in Poland. The database was described previously [28]. Although it is an open database and statistics for a given calendar year are compiled during that year, some records can be added to the database later on. It contains statistics regarding all publicly financed surgeries (almost all performed in the whole country, without minor procedures funded from private sources) including 1 day procedures and procedures in private hospitals (where most of the procedures are financed by the NHF).

The term “in-hospital death” was characterized as death that occurred within the period of hospitalization during which a surgery took place. The NHF database recorded such instances as “death” but a fatality which occurred after discharge was not recorded. A crude mortality rate was characterized as the number deaths resulting from surgical hospitalizations during a given year divided by total number of surgical hospitalizations that occurred in the same year.

The procedures were divided according to the diagnosis-related groups (DRG) catalog into neurosurgery, gastrointestinal tract, heart and circulation, vascular, respiratory system, liver, biliary tract, pancreas and spleen, bones and muscles, skin and mammary gland, genitourinary tract (urological), endocrinological, head and neck, female genital tract, eye surgeries, and injuries. The following risk factors of mortality were assessed: DRG category, age (patients were distributed among age groups: 18–24; 25–34; 35–44; 45–54; 55–64; 65–74; 75–84; 85–94 and ≥ 95 years), sex, type of surgery (elective, urgent or emergency, where an emergency patient was defined as a patient brought to the hospital by an ambulance), length of stay (arbitrarily divided into the following periods: 0, 1, 2–4, 5–7, 8–14, 15–21, 22–30, 31–60, ≥ 61 days), week day of admission and whether the day of admission was a bank holiday, as well as category of the hospital (teaching, regional, district or city, private).

### Statistical analysis

The multivariable logistic regression model which combined all of the previously mentioned predictors was implemented to calculate the adjusted odds ratios (ORs). To handle the quasi‐complete separation of data, the Firth correction was applied. The following tests were performed to assess model adequacy (assuming significancy at *P* value level of less than 0.05): the likelihood ratio test for the joint significance of predictors, the Wald χ^2^ test for single‐predictor significance, as well as the Hosmer–Lemeshow and Osius–Rojek goodness of fit tests.

Odds ratios greater than one indicated a higher risk of in‐hospital fatality, and those below one a lower in‐hospital fatality risk in the investigated group. Furthermore, 95% CIs were calculated for ORs. The Wald χ^2^ test was performed to verify whether ORs were different than 1. The analysis was conducted using the SAS system (SAS Institute Inc., Cary, North Carolina, United States) [29].

## Results

In 2014, there were 2,800,069 hospitalizations related to surgical procedures (women: 1,739,693, men: 1,060,376) and 22,439 of them resulted in deaths; therefore, the in-hospital mortality rate was 0.8% (women 0.6%, men 1.13%). Female gender was associated with a lower mortality risk (OR 0.82; CI 0.80–0.85; *p* < 0.0001).

**Fig. 1:**
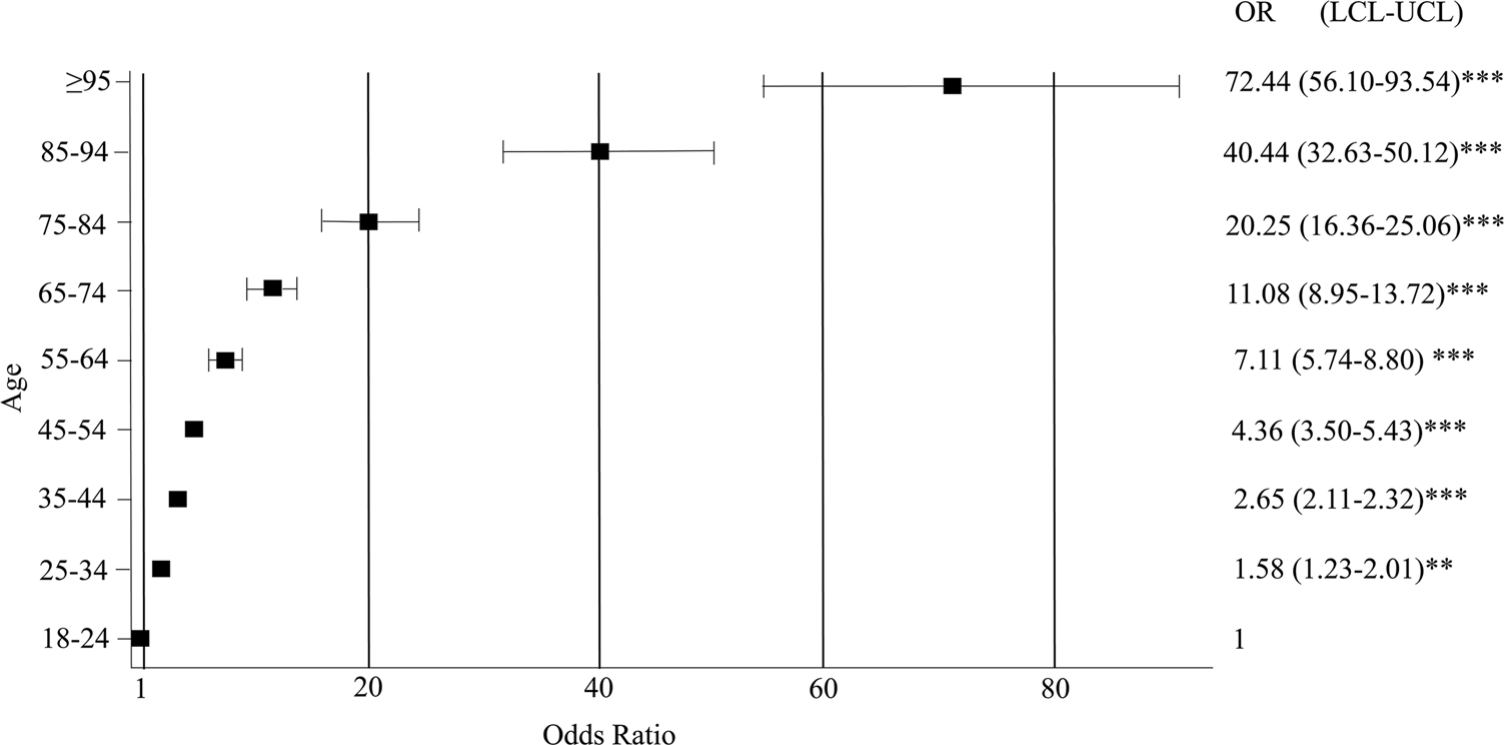
In-hospital surgical mortality according to age. ***p < 0.0001 **p < 0.001. OR odds ratio, LCL lower confidence limit, UCL upper confidence limit

The rate of mortality was different in the particular age groups. It was equal 0.06% in 18–24 years group, 1.03% in 65–74, 1.97% in 75–84, 5.05% in 85–94 and 11.44% in 95+years groups. The in-hospital-death OR (odds ratio) was 72.4-fold higher in the oldest group as compared with the youngest one (Fig. 1).

The highest mortality rate involved patients with injuries (24.5%) followed by neurological and gastrointestinal surgeries (4.90% and 2.30%, respectively). Mortality rates above 1% were observed for vascular, heart and circulation, respiratory system and liver, biliary tract, pancreas, and spleen surgeries (1.64%, 1.62%, 1.50%, and 1.19%, respectively). Adjusted ORs for in‐hospital mortality in surgical departments based on the DRG category are shown in Table 1.

**Table 1:** Odds ratios for in‐hospital mortality in surgical departments based on the DRG category (compared with the vascular category)

DRG	Coefficient	SE	WaldChiSq	*P*-value	OR	CI
Injuries	1.22	0.16	60.64	< 0.0001	3.39	2.49–4.60
Neurosurgery	0.80	0.03	529.89	< 0.0001	2.23	2.08–2.38
Gastrointestinal tract	0.30	0.03	117.41	< 0.0001	1.35	1.28–1.42
Respiratory system	0.23	0.05	20.18	< 0.0001	1.26	1.14–1.39
Liver, biliary tract, pancreas and spleen	−0.44	0.04	142.34	< 0.0001	0.65	0.60–0.69
Heart and circulation	−0.62	0.03	463.02	< 0.0001	0.54	0.51–0.57
Endocrinological	−0.88	0.17	27.16	< 0.0001	0.42	0.30–0.58
Skin and mammary gland	−1.04	0.05	377.34	< 0.0001	0.35	0.32–0.39
Bones and muscles	−1.33	0.03	1743.55	< 0.0001	0.27	0.3–0.28
Head and neck	−1.75	0.09	351.48	< 0.0001	0.17	0.15–0.21
Genitourinary (urology)	−1.87	0.06	1084.25	< 0.0001	0.15	0.14–0.17
Female genital tract	−3.06	0.10	999.81	< 0.0001	0.05	0.04–0.06
Eye surgeries	−5.16	0.26	394.86	< 0.0001	0.01	0.00–0.01

*SE* standard error; *CI* confidence interval; *Coefficient* the estimated log odds ratio between given DRG and a base DRG

In 2014, there were 1,811,845 elective admissions, 881,330 urgent admissions and 106,894 emergency admissions to surgical wards. The mean in-hospital mortality rate was 0.18% in the elective admission group, 1.41% in the urgent-admission group and 6.34% in the emergency admission group. The mortality risk was significantly higher in case of urgent (OR 6.90; CI 6.62–7.20; *p* < 0.0001) and emergency admissions (OR 15.69; CI 14.94–16.46; *p* < 0.0001).

The database comprised of 522,596 hospitalizations in teaching hospitals, 978,716 in district or city hospitals, 771,861 in regional hospitals and 526,896 in private ones. The mortality rate assessed for teaching hospitals was 0.89%, in district or city hospitals 0.81%, in regional hospitals 1%, whereas in private hospitals 0.42%. The risk of death in district or city hospitals and in regional hospitals was comparable to teaching hospitals. Compared to teaching hospitals, the risk of death in private hospitals was significantly lower (OR 0.64; CI 0.60–0.67; *p* < 0.0001).

**Fig. 2:**
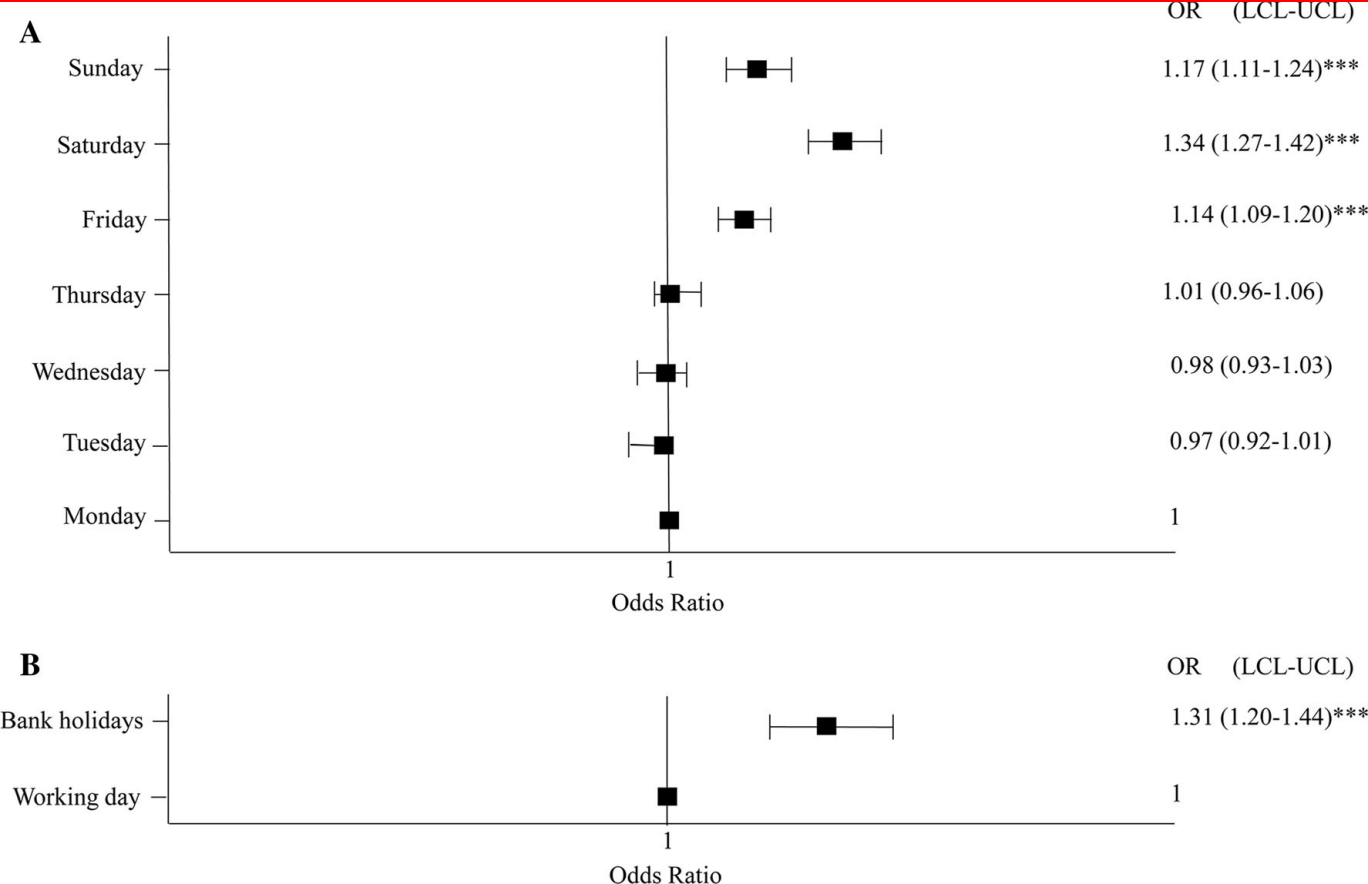
**a**. In-hospital surgical mortality according to the day of admission. **b**. In-hospital surgical mortality according to bank holiday. ***p < 0.0001. OR odds ratio, LCL lower confidence limit, UCL upper confidence limit

According to the database, 408,957 admissions occurred on the weekend or another non-working day, and the mean in-hospital mortality in this group was 1,37%. On working days there were 2,424,499 admissions with the mean in-hospital mortality rate of these patients being 0,72%. The number of admissions decreased gradually from monday to saturday and again increased slightly on sunday. The number of admissions on mondays was about 5 times higher than on saturdays when the number of admissions was the lowest. Admission during the weekend or bank holidays was a significant predictor of in-hospital death and the “weekend” effect appears to begin on Friday (Fig. 2a, b). Despite the lower number of all types of hospital admissions during the weekends and bank holidays, the death rate was higher for almost all of them compared to the regular workdays. This pattern was similar, although slightly different for planned, urgent and emergency admissions (Table 2).

**Table 2:** In-hospital surgery-related mortality for particular day and type of admission

Type of admission	Day	Number of hospitalizations	Number of deaths	Mortality rate (%)	*p*-value
Planned	Mon-Thu	1,302,398	2363	0.18	0.1
	Fri-Sun	509,447	911	0.18	
	Regular	1,800,361	3242	0.18	< 0.001
	Bank holiday	11,484	32	0.28	
Urgent	Mon-Thu	558,305	7393	1.32	< 0.001
	Fri-Sun	323,025	4991	1.55	
	Regular	862,750	12,075	1.40	< 0.05
	Bank holiday	18,580	309	1.66	
Emergency	Mon-Thu	62,804	3896	6.20	< 0.05
	Fri-Sun	44,090	2885	6.54	
	Regular	103,571	6547	6.32	0.09
	Bank holiday	3323	234	7.04	

**Fig. 3:**
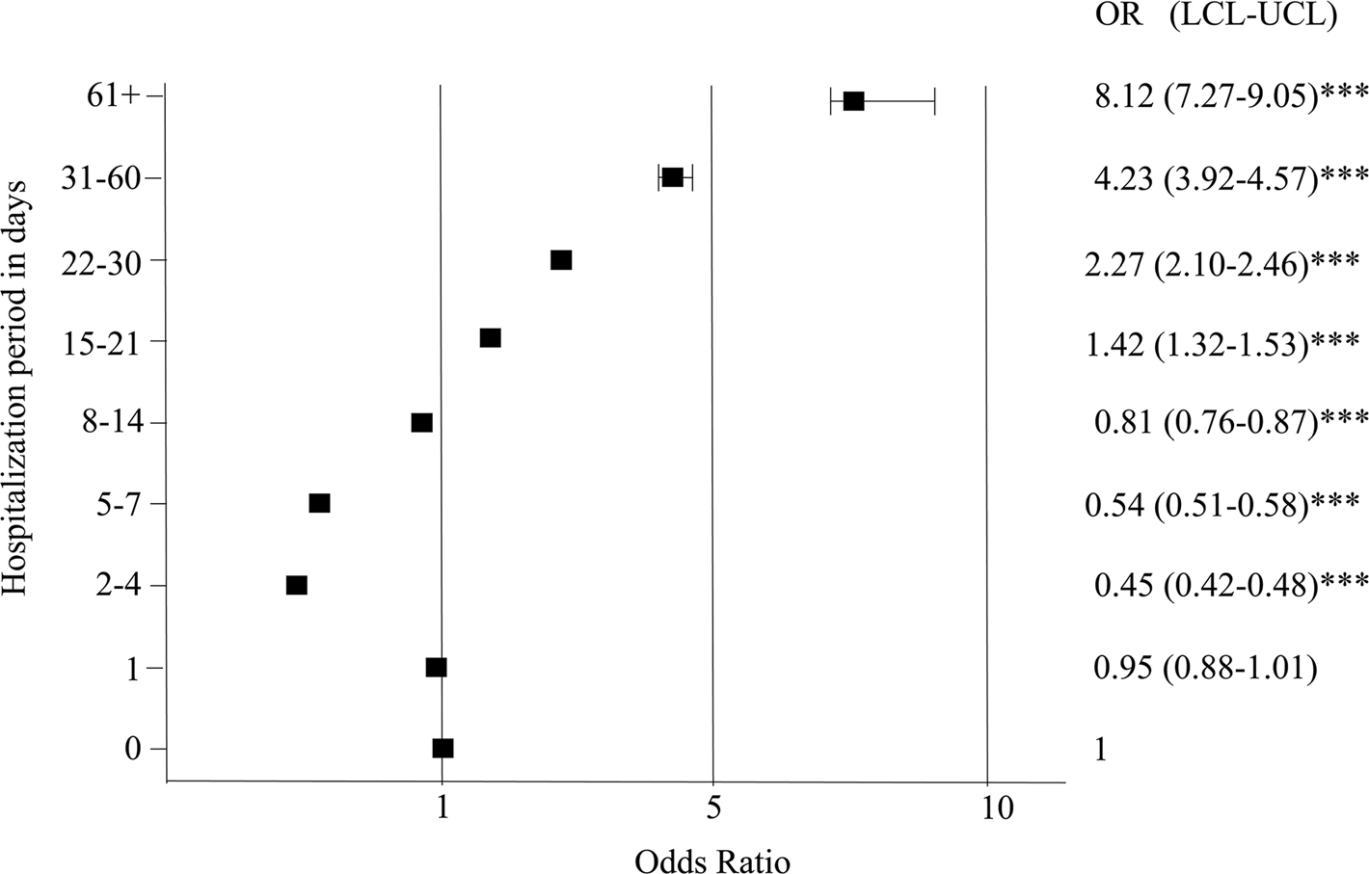
In-hospital surgical mortality according to the duration of hospitalization. ***p < 0.0001. OR odds ratio, LCL lower confidence limit, UCL upper confidence limit

Additionally, the death rate was related to the hospitalization length. It was similar for 0 days and one day hospitalizations, whereas it was decreased (compared to 0 day) for periods of 2–4, 5–7, and 8–14 days, and increased (compared to 0 day) for longer hospitalizations. The highest in-hospital mortality rate was for hospitalizations lasting longer than 60 days (Fig. 3). It has to be mentioned however, that if the type of admission was taken into account, the highest death rate was observed for those patients, who were admitted to the hospital in an acute condition (emergency admission) (Table 3).

**Table 3:** In-hospital surgery-related mortality according to duration of hospitalization and admission type

Number of days of the hospitalization	Type of admission	Number of hospitalizations	Number of deaths	Mortality rate
< 1	Planned	498,276	52	0.01%
	Urgent	73,676	795	1.1%
	Emergency	2880	725	25.2%
1	Planned	264,380	115	0.04%
	Urgent	83,621	1136	1.4%
	Emergency	6231	833	13.4%
2–4	Planned	756,167	570	0.1%
	Urgent	393,590	2406	0.6%
	Emergency	34,329	1449	4.2%
5–7	Planned	194,527	460	0.2%
	Urgent	174,313	1623	0.9%
	Emergency	25,165	893	3.5%
8–14	Planned	126,726	794	0.6%
	Urgent	118,256	2636	2.2%
	Emergency	25,431	1306	5.1%
15–21	Planned	24,005	437	1.8%
	Urgent	28,976	1434	4.9%
	Emergency	8021	636	7.9%
22–30	Planned	8284	319	3.9%
	Urgent	12,404	997	8.0%
	Emergency	3633	416	11.5%
31–60	Planned	5081	432	8.5%
	Urgent	9015	1164	12.9%
	Emergency	2775	484	17.4%
61+	Planned	986	183	18.6%
	Urgent	2116	383	18.1%
	Emergency	745	188	25.2%

## Discussion

In this work we analyze predictors of in-hospital mortality in surgery patients. Interestingly, we show that older age, male gender, surgery type, urgent and emergency admission, admission on the weekend or bank holiday, duration of hospitalization were factors associated with a greater mortality. This retrospective cohort study is strengthened by the fact that it includes a high number of cases retrieved from a large database covering almost all cases of surgery performed in adults nationwide. Additionally, the classification of an outcome of every hospitalization and its mandatory reporting to the NHF database enhances the reliability of the results.

It is common knowledge that in-hospital mortality increases with age, which is associated with more comorbidities, frailty, and decreased adaptation to various injuring factors. The results of this study are consistent with other observations [24, 29–31].

The importance of sex as an in-hospital risk factor in the general population undergoing surgery has been poorly studied. Here, we analyse all kinds of surgical procedures together and find that female patients were less likely to die than male patients. There are some papers that show a similar trend [32], but others found no differences between the sexes with respect to mortality [33]. Therefore, this issue requires more detailed studies in the future.

Our study demonstrated that certain types of surgeries are associated with an increased in-hospital mortality. The highest mortality included surgical patients with injuries (24.5%) followed by neurosurgeries and gastrointestinal surgeries (4.90 and 2.30%, respectively). Mortality rates above 1% were observed for vascular, heart and circulation, respiratory system and liver, biliary tract, pancreas, and spleen surgeries. Similarly, Shidara et al. demonstrated in a retrospective cohort analysis of a Japanese university hospital that surgical procedures like neurosurgery, cardiovascular surgery, gastrointestinal tract surgery, catheter intervention and procedures of emergency medicine have crude mortality greater than 1% and classified them as a high-risk surgical group. Also, some operations are associated with significant inpatient mortality, but high-risk surgery is not well defined and debate on this topic is still open [17].

The mode of admission is a crucial predictor of mortality. We demonstrated that the risk of death has been 6.9-fold and 15.69-fold greater for urgent and emergency admissions (*p* < 0.0001) compared to elective ones, respectively. The results of this analysis are in line with many other studies [34–36]. Patients undergoing urgent/emergency surgeries appear to have a higher comorbidity burden and a lower preoperative functional status and therefore have poorer outcomes.

The outcome of hospitalization can be affected by the “weekend effect”. A recent systematic review and meta-analysis of cohort studies [37] integrated statistics from a set of 29 investigations which including over 8 million patients, in order to identify the impact of the weekend effect on surgical care. The authors confirmed that the risk of postoperative death increases as the day of admission approaches the weekend. We observed the same trend. Additionally, mortality on weekends and bank holidays was higher compared to the regular workdays. The only clear exception is that planned admissions during weekends do not result in greater mortality as compared with planned admissions during the working days. This is likely because patients that get admitted during the weekend will have their planned surgery on the following working day.

A reduced number of hospital staff during the weekend or bank holidays may result in shortfalls in care. If this is the case, better redistribution of available staff resources to cover the potential gaps in care may be needed to help reduce this mortality rate.

Ogola et al. demonstrated, that the mortality in surgical units may be related to hospital volume, location of the hospital (urban or rural), type of the hospital (teaching or not teaching), and hospital ownership [38]. In our study, we considered the hospital type, however, our division into hospital subtypes was different than in Ogola’s paper and we had no data regarding the hospital volume. In our study, the risk of death in district or city hospitals and regional hospitals was comparable to that of teaching hospitals.

Mortality rate seems to be lower in private hospitals. A private hospital in Poland means that the state is not its owner, but many surgical procedures in such hospitals are financed by the NHF. It should be emphasized that the types of surgeries performed in private and public hospitals are different. Private hospitals perform only planned, low-risk procedures, and the vast majority of the high-risk procedures, associated with higher mortality are performed in public hospitals. Private hospitals admit low-severity patients are often specialized in certain procedures and only those procedures are performed. Similar situations are also observed in other countries [39, 40]. Given the reasons mentioned above, we cannot conclude that hospitalization in a private hospital is a predictor of lower mortality.

The relationship between the length of stay and in-hospital mortality should also be emphasized. A longer postoperative length of stay (especially more than 15 days) is associated with a higher in-hospital mortality rate. This is consistent with most publications [2, 41] and follows from patients’ comorbidities and complications. The relatively high-mortality rate observed for patients hospitalized for 0 and 1 day was probably associated with a higher percentage of emergency admissions (Table 1). Interestingly, in a Swedish analysis performed by Nordström et al., a shorter duration of hospitalization after a hip fracture was related to an increased risk of dying after discharge, but only when the duration of stay was 10 days or shorter [42].

Our study has some limitations. First, some procedures financed from private sources are not captured by the NHF database. However, they comprise only a small fraction of all surgeries performed in Poland. Secondly, the NHF database does not capture post-discharge mortality. Nevertheless, in-hospital mortality rate is a recognized measure of health care quality, and data regarding exclusively this measure have been published in some of the most important clinical journals [34].

## Conclusion

In conclusion: age, male gender, emergency admission, weekend or bank holiday admission, length of stay in the hospital are factors associated with greater mortality in surgical units. This population of patients require increased monitoring and special attention. Better redistribution of staffing on weekend and bank holidays can help lower the mortality risks but this issue requires further research.

This analysis could also be the first step to create Hospital Standardized Mortality Ratio (HSMR) for Poland. An HSMR can be used to estimate a hospital’s mortality rate and track performance thereby helping to improve safety and quality of care.
